# Supplementary material for: Monomethyl auristatin antibody and peptide drug conjugates for trimodal cancer chemo-radio-immunotherapy
Source: Nat Commun. 2022 Jul 5;13:3869. doi: 10.1038/s41467-022-31601-z (PMC9256669; doi:10.1038/s41467-022-31601-z)
Supplement: Supplementary file 1 — Supplementary Information [file 41467_2022_31601_MOESM1_ESM.pdf]

Monomethyl auristatin antibody and peptide drug conjugates for trimodal cancer chemo-radio-immunotherapy

Dina V. Hingorani[1#], Michael M. Allevato[#2], Maria F. Camargo[1], Jacqueline Lesperance[1], Maryam A. Quraishi[1], Joseph Aguilera[1], Ida Franiak-Pietryga[1], Daniel J Scanderbeg[1], Zhiyong Wang[2], Alfredo A. Molinolo[3,4], Diego Alvarado[5], Andrew B. Sharabi[1,4], Jack D. Bui[3,4], Ezra E.W. Cohen[4,6], Stephen R. Adams[2], J. Silvio Gutkind[2,4], Sunil J. Advani[1,4*]

[1]Department of Radiation Medicine and Applied Sciences, University of California San Diego, La Jolla, CA 92093, USA
[2]Department of Pharmacology, University of California San Diego, La Jolla, CA 92093, USA
[3]Department of Pathology, University of California San Diego, La Jolla, CA 92093, USA
[4]UC San Diego, Moores Cancer Center, La Jolla, CA 92093, USA
[5]Celldex Therapeutics, Hampton, NJ 08827, USA
[6]Department of Medicine, Division of Hematology and Oncology, University of California San Diego, La Jolla, CA 92093, USA

[#] These authors contributed equally

[*]Corresponding Author
Sunil J. Advani
Department of Radiation Medicine and Applied Sciences
Moores Cancer Center
University of California San Diego
3855 Health Sciences Drive, MC 0843
La Jolla, CA 92093-0843
Phone: 858-822-6046
Fax: 858-822-5568
Email: sjadvani@ucsd.edu

Supplementary Information

Supplementary Figure 1

a

Pathway	# of Genes	Undirected Enrichment Score	Directed Enrichment Score
Angiogenesis	35	2.7066	1.8412
Matrix Remodeling and Metastasis	61	2.3587	1.1775
Interferon Signaling	61	1.5239	0.9986
Apoptosis	38	2.3071	0.9856
Cytotoxicity	50	1.637	0.8779
Lymphoid Compartment	89	1.1946	0.8253
Antigen Presentation	57	1.2276	0.8121
Myeloid Compartment	77	1.2711	0.1922
NF-kappaB Signaling	36	1.6264	-0.7242
Cell Proliferation	49	2.1596	-0.7774
Immune Cell Adhesion and Migration	85	1.8838	-1.0133
Notch Signaling	26	2.5438	-1.0152
PI3K-Akt	101	2.7297	-1.358
Cytokine and Chemokine Signaling	106	1.9551	-1.4236
MAPK	77	2.756	-1.437
JAK-STAT Signaling	59	2.5395	-1.657
Epigenetic Regulation	22	2.4405	-1.7988
Costimulatory Signaling	84	2.2732	-1.9366
DNA Damage Repair	32	2.4043	-1.9501
Hedgehog Signaling	19	3.1229	-2.2414
TGF-beta Signaling	20	3.339	-2.2971
Wnt Signaling	34	3.1574	-2.3166
Metabolic Stress	82	2.8728	-2.3196
Hypoxia	42	2.9454	-2.4508
Autophagy	23	3.5536	-3.279

b

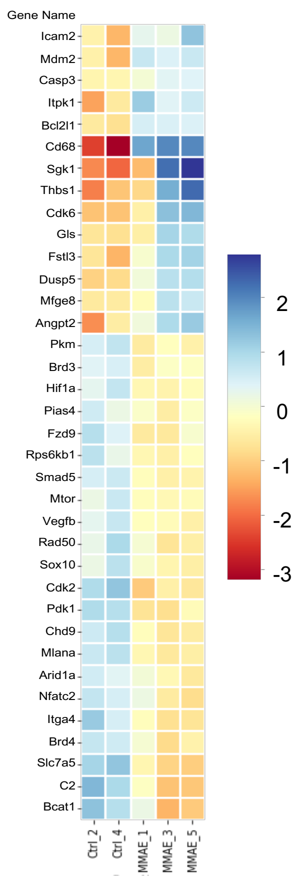

Supplementary Figure 1: Gene expression analysis of MMAE treated cells. B16 cells exposed to 5 nM MMAE for 72 hrs. RNA collected and analyzed using murine NanoString PanCancer IO 360 Panel. **a)** Gene set analysis with calculated global significance scores and directed significance scores. **b)** Heatmap depicting differential gene expression of 36 genes significantly altered between untreated control and MMAE treated cells. Source data are provided in Source Data file under tab labeled Figure 1b.

Supplementary Figure 2

a

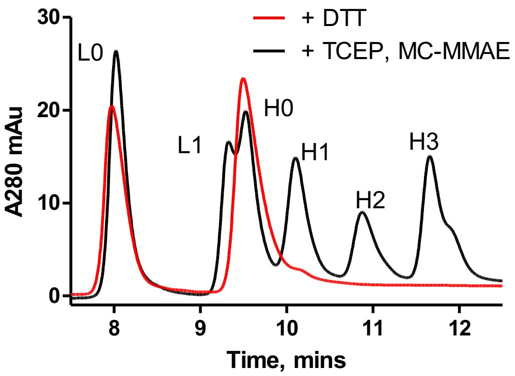

Peak	MW (found)	Assignment
L0	22671	Light chain (LC)
L1	24004	LC-(MMAE)
H0	50307	Heavy Chain (HC)
H1	51623	HC-(MMAE)
H2	52937	HC-(MMAE)$_2$
H3	54249	HC-(MMAE)$_3$

b

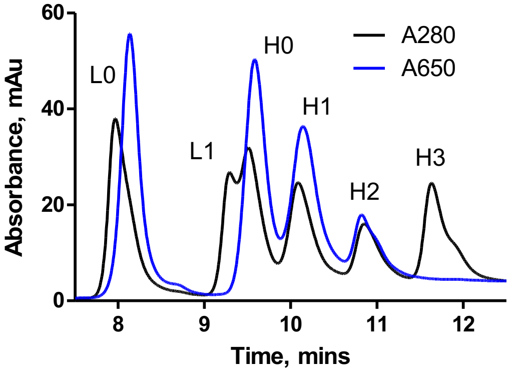

Peak	MW (found)	Assignment
L0	22699, 23473	LC, LC-Cy5
L1	24004	LC-(MMAE)
H0	50328, 51107	HC, HC-Cy5
H1	51649, 52412	HC-(MMAE), HC-(MMAE)-Cy5
H2	52934, 53736	HC-(MMAE)$_2$, HC-(MMAE)$_2$-Cy5
H3	54251	HC-(MMAE)$_3$

c

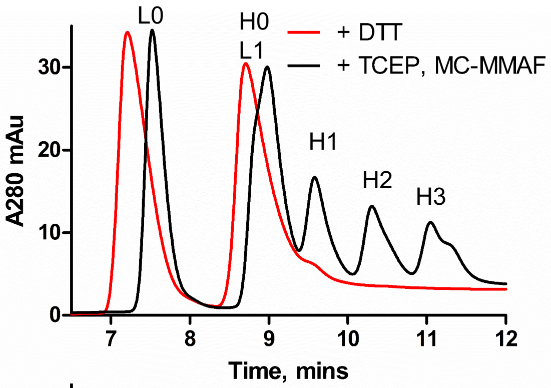

Peak	MW (found)	Assignment
L0	22678	Light chain (LC)
L1	24003	LC-(MMAF)
H0	50243	Heavy Chain (HC)
H1	51581	HC-(MMAF)
H2	52909	HC-(MMAF)$_2$
H3	54244	HC-(MMAF)$_3$

d

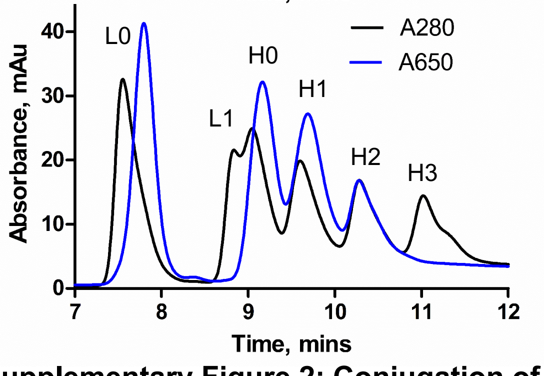

Peak	MW (found)	Assignment
L0	22680, 23459	LC, LC-Cy5
L1	24010	LC-(MMAF)
H0	50231, 51036	HC, HC-Cy5
H1	51604, 52374	HC-(MMAF), HC-(MMAF)-Cy5
H2	52896, 53686	HC-(MMAF)$_2$, HC-(MMAF)$_2$-Cy5
H3	54248	HC-(MMAF)$_3$

Supplementary Figure 2: Conjugation of auristatins and Cy5 to CDX3379. a) Reverse-phase protein chromatograms and electro-spray mass spectroscopy analysis of CDX-3379 after reduction with excess DTT or with 4 equivalents of TCEP and subsequent reaction with 4 equivalents of MC-VC-PABC-MMAE and **b)** 2 equivalents of Cy5-maleimide, followed by gel filtration and concentration. Samples were treated with 50 mM DTT at 37˚C for 15 min prior to analysis. Mass calculated from 12+ and 28+ peaks for light and heavy chains respectively. **c)** Reverse-phase protein chromatograms and electro-spray mass spectroscopy analysis of CDX-3379 after reduction with excess DTT or with 4 equivalents of TCEP and subsequent reaction with 4 equivalents of MC-VC-PABC-MMAF and **d)** 2 equivalents of Cy5-maleimide, followed by gel filtration and concentration. Samples were treated with 50 mM DTT at 37˚C for 15 min prior to analysis. Mass calculated from 12+ and 30+ peaks for light and heavy chains respectively. For CDX3379-MMAE analysis in a & b, plots are representative of 15 independent syntheses. For CDX3379-MMAF analysis in c & d, plots are representative of 3 independent syntheses.

Supplementary Figure 3

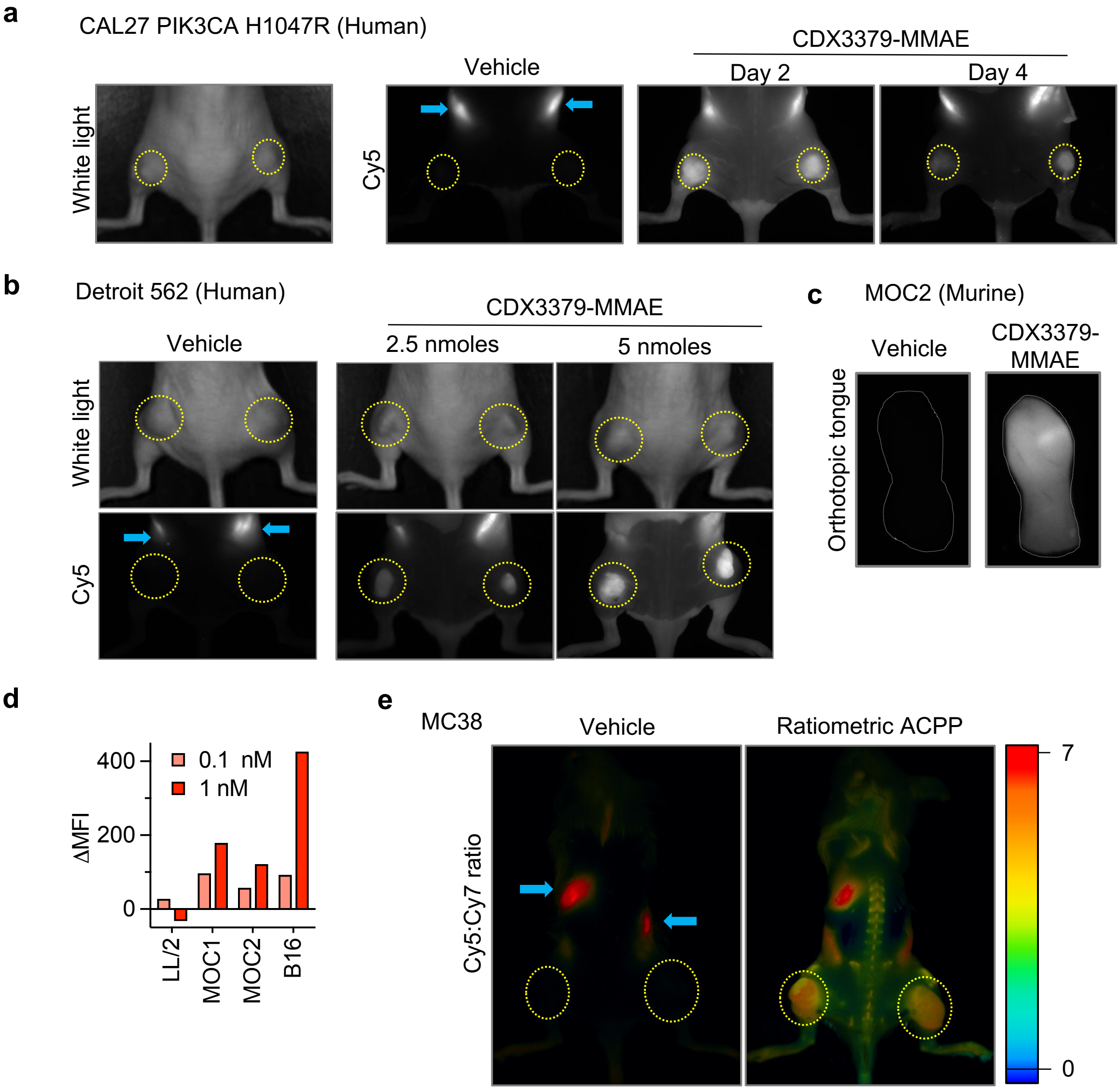

Supplementary Figure 3: In situ whole mouse optical imaging of fluorescently labeled ADC or ACPP. **a)** Cy5 localization of CDX3379-MMAE ADC in CAL27 human tumor xenografts (tumor locations circled yellow). Mice i.v. injected with 2.5 nmoles Cy5 labeled CDX3379-MMAE. White light imaged mouse on far left. Cy5 captured images at indicated time points. Images representative of 3 mice/group. **b)** Cy5 localization of CDX3379-MMAE ADC in Detroit 562 human tumor xenografts (tumor locations circled yellow). Mice i.v. injected with 2.5 or 5 nmoles Cy5 labeled CDX3379-MMAE and imaged 2 days post injection. White light images (top) and Cy5 images (bottom) captured. Images representative of 2 mice/group. **c)** Cy5 localization of CDX3379-MMAE in syngeneic orthotopic tongue tumors. 2.5 nmoles of Cy5 labeled CDX3379-MMAE injected into MOC2 tumor bearing mice. Tumor bearing tongues resected and imaged for Cy5 (white signal) at 48 hr. Imaged tongue delineated by dashed gray line. Images representative of 3 mice/group. **d)** Dose dependent cell surface binding of CDX3379-MMAE conjugates. Murine cancer cells incubated on ice with Cy5 labeled ADC, bound Cy5 signal measured by flow cytometry. Mean fluorescent intensity measured and subtracted from baseline fluorescence of saline incubated cells for each cell line. Data representative of 3 independent experiments. **e)** Mice with subcutaneous murine MC38 tumors (tumor locations circled yellow) i.v. injected with 10 nmoles ratiometric ACPP. *In situ* whole mouse imaging for Cy5 and Cy7 direct fluorescence with Cy5:Cy7 emission ratio determined, pseudocolor scale bar shown far right. Gut auto-fluorescence indicated by blue arrows. Images representative of 2 mice/group. Source data are provided in Source Data file.

Supplementary Figure 4

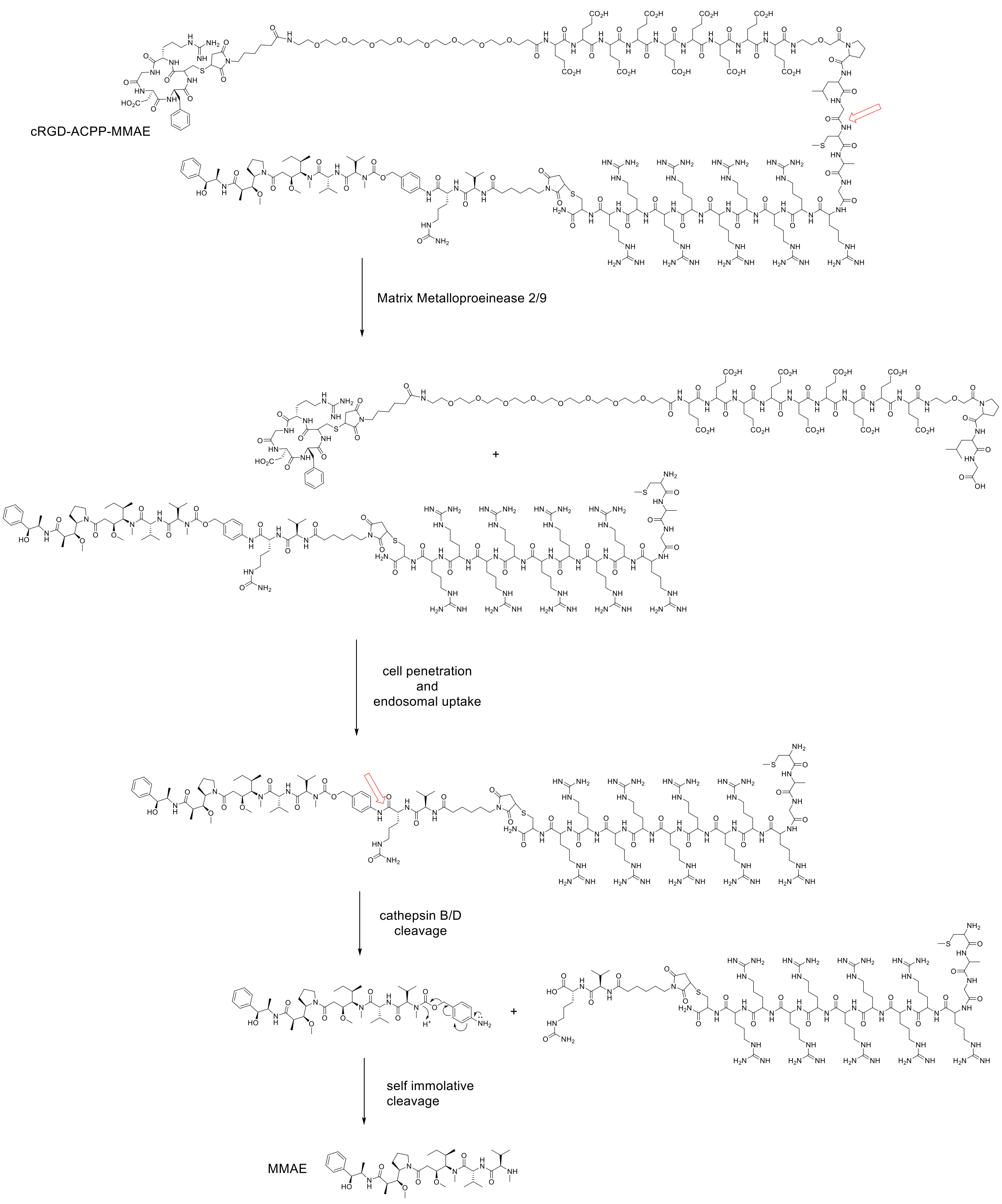

Supplementary Figure 4: Schema for unmasking ACPP-MMAE and drug release. Extracellular matrix metalloproteinase 2/9 cleavage of ACPP-MMAE releases cell penetrating peptide linked MMAE which is then internalized into cells within endosomes. Endosomal cathepsin B/D cleaves the valine-citrulline dipeptide linker of MC-VC-PABC followed by PABC self-immolation and intracellular release of free MMAE.

Supplementary Figure 5

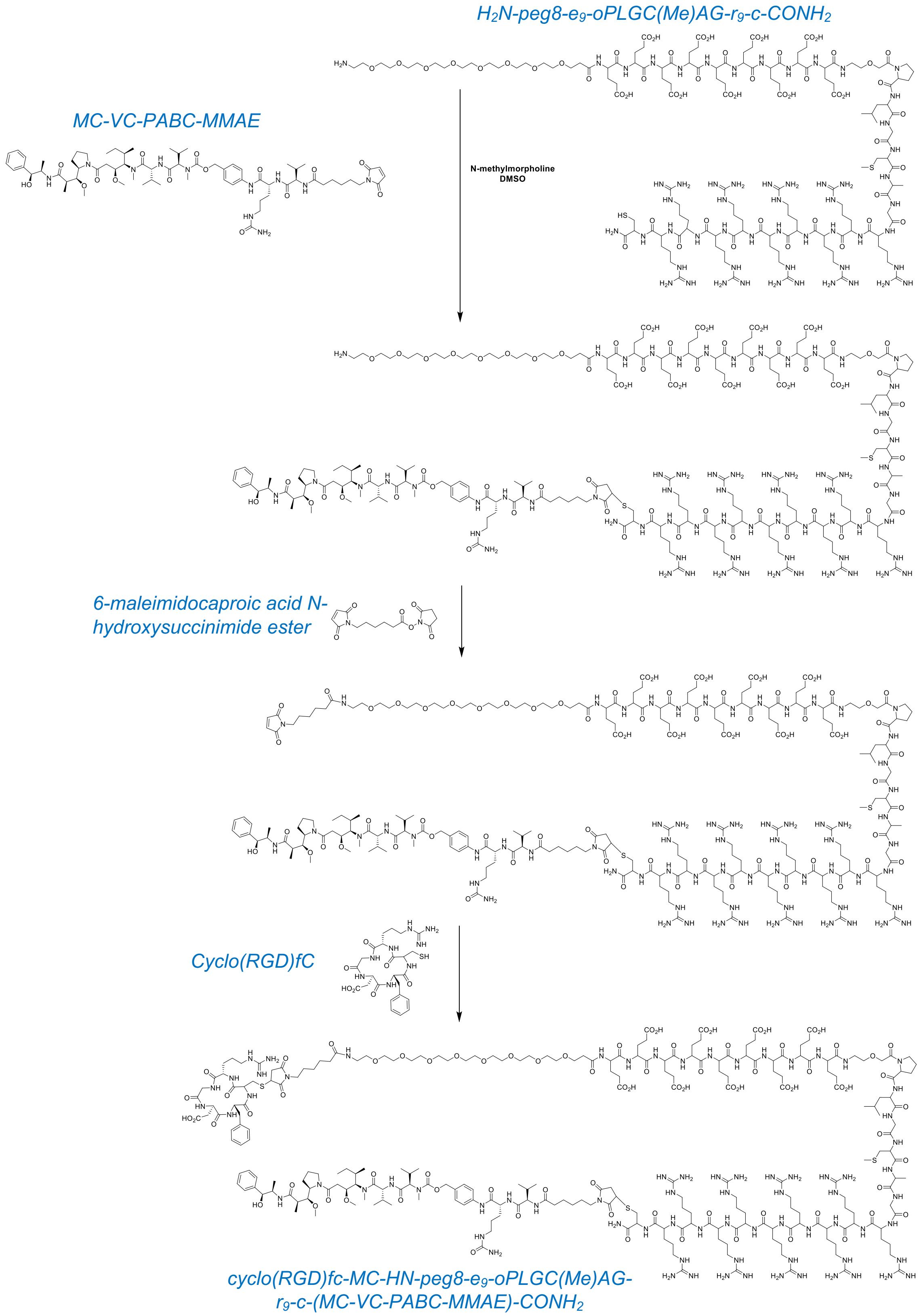

Supplementary Figure 5: Synthetic scheme for activatable cell penetrating peptide conjugated to MMAE. MMAE bound to MC-VC-PABC linker reacted with ACPP. cRGD reacted with MMAE conjugated ACPP to yield co-targeted ACPP-MMAE (cyclo(RGD)fc-MC-HN-peg8-e$_9$-oPLGC(Me)AG-r$_9$-c-(MC-VC-PABC-MMAE)-CONH$_2$).

Supplementary Figure 6

a

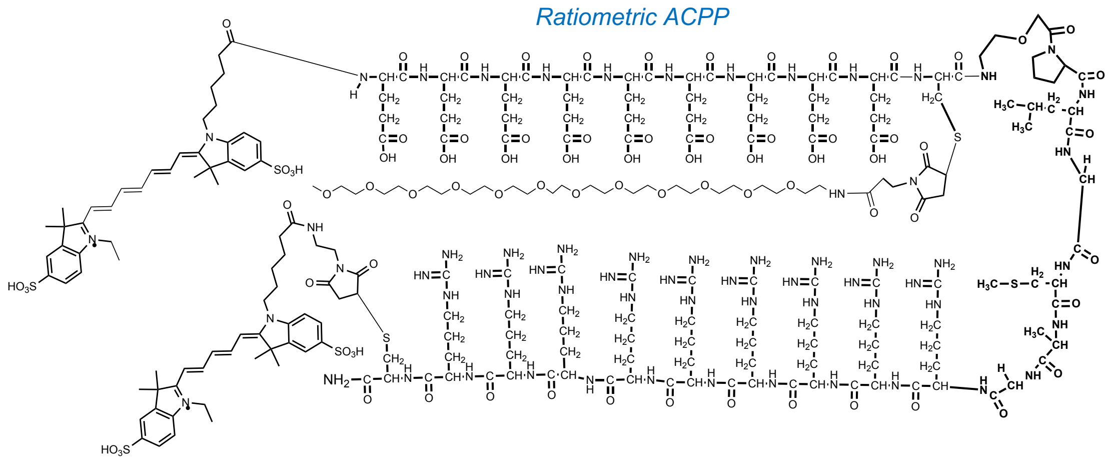

b

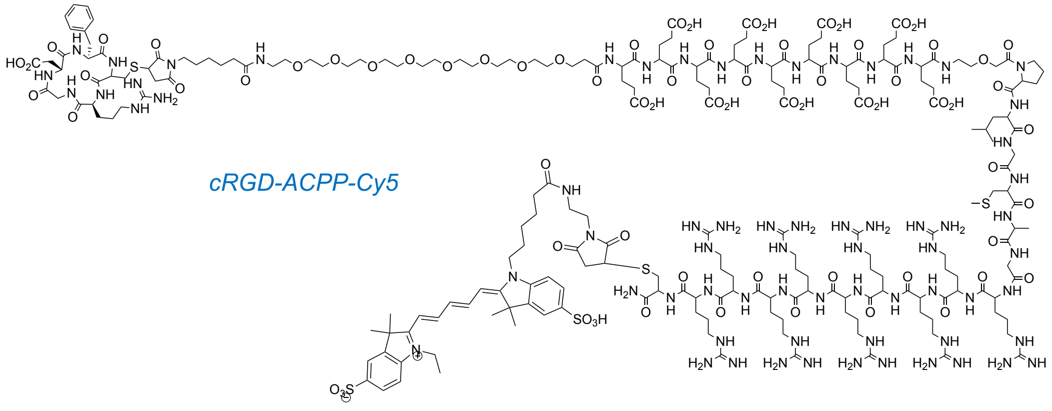

Supplementary Figure 6: Chemical structures of synthesized fluorescently labeled activatable cell penetrating peptides. a) Ratiometric ACPP with Cy5 and Cy7 attached. **b)** Co-targeted cRGD-ACPP-Cy5.

Supplementary Figure 7

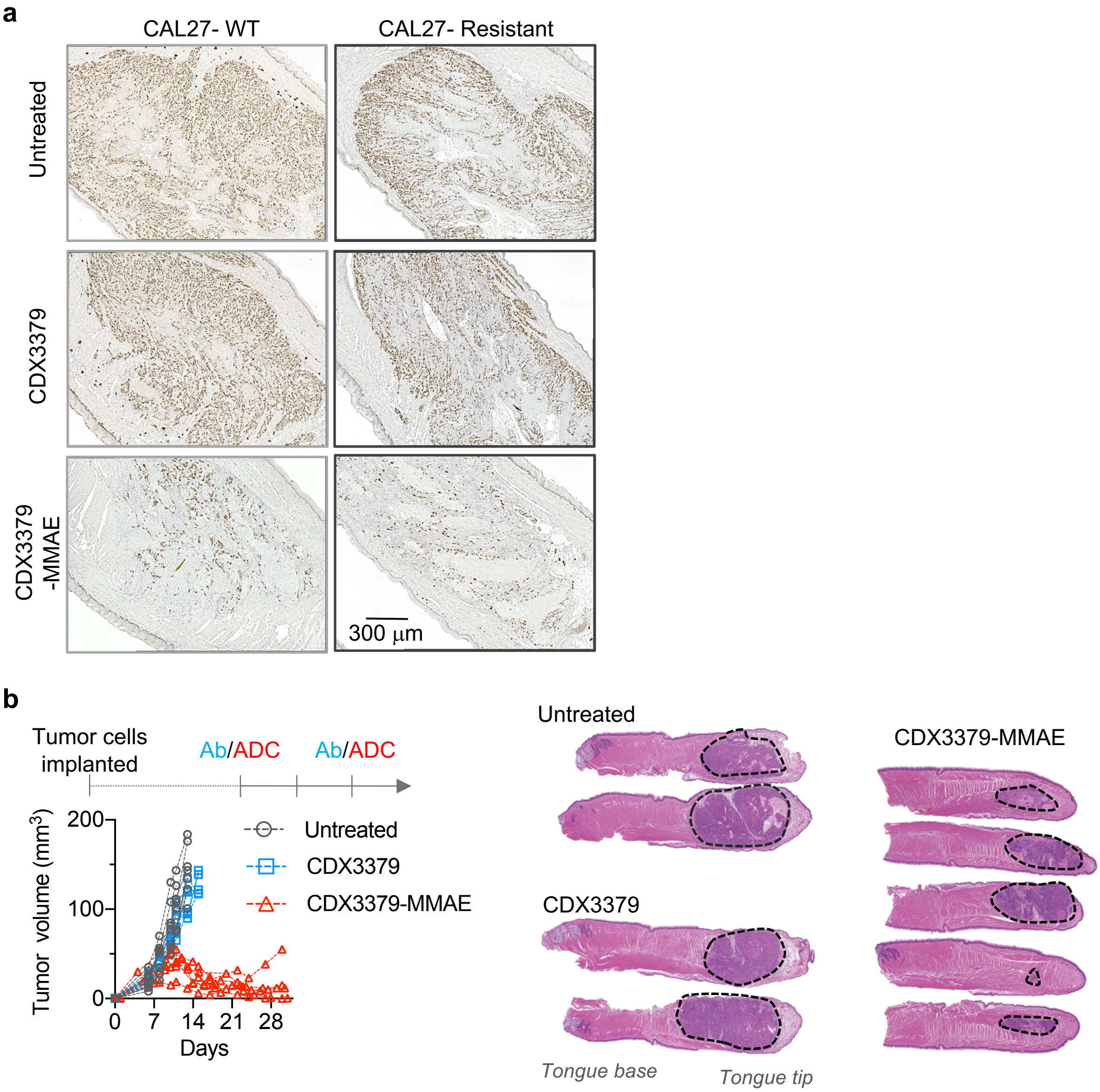

Supplementary Figure 7: CDX3379-MMAE ADC efficacy in tumor xenografts. CAL27 parental (WT) or HER3 resistant (PIK3CA H1047R) tumor xenografts established in murine tongues on day 0. **a)** On day 7, mice i.v. injected with 2.5 nmoles CDX3379 antibody or CDX3379-MMAE ADC. On the last day of tumor measurement (day 16 for WT, day 14 for PIK3CA H1047R tumors), tongues resected, fixed and sectioned. Tissues stained for Ki-67 (Abcam, ab16667) by IHC and counterstained with hematoxylin. Data is representative of 6 independent tumors per tumor cell line and treatment. **b)** Mice with orthotopic tongue CAL27 PIK3CA H1047R tumors were untreated (n=8 tumors) or i.v. injected with 2.5 nmoles CDX3379 antibody or CDX3379-MMAE ADC on day 6 and day 8 (n= 6 tumors/group). On the last day of tumor measurements, tongues were resected, fixed, longitudinally sectioned and H&E stained. Source data are provided in Source Data file.

Supplementary Figure 8

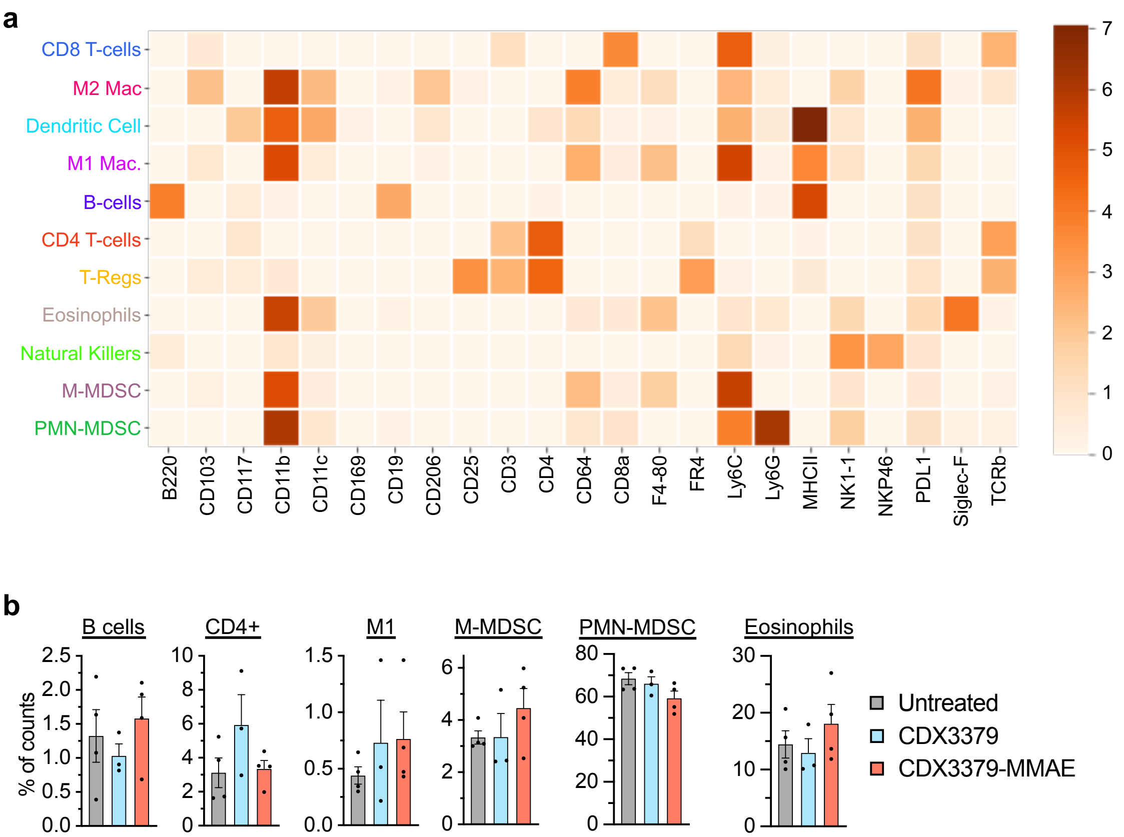

Supplementary Figure 8: CyTOF analysis of tumor immune infiltrates. a) Heatmap of the median marker intensities of the 23 lineage markers across 11 cell populations identified with opt-SNE. **b)** MOC2 murine tumors orthotopically implanted in tongues on day 0. On day 7, mice i.v. injected with 2.5 nmoles CDX3379 antibody or CDX3379-MMAE ADC. Tumors harvested on day 11, analyzed by CyTOF, visualized by opt-SNE. Mean percent of immune cell types per sample count ± SEM. Untreated and CDX3379-MMAE, n=4 tumors; CDX3379, n=3 tumors. No statistically significant differences were noted in these immune cell types. Source data are provided in Source Data file.

Supplementary Figure 9

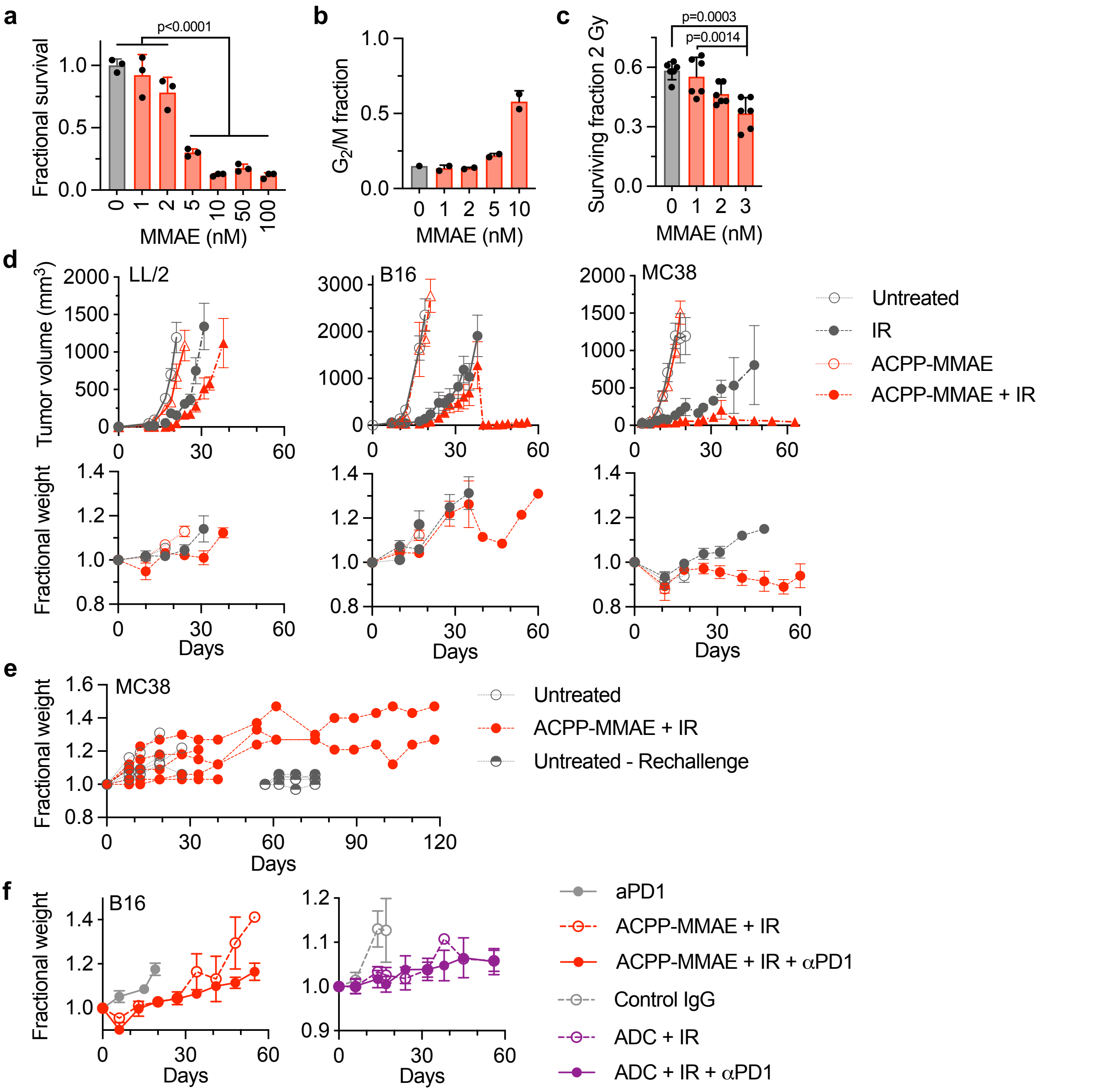

Supplementary Figure 9: Safety and efficacy of MMAE with IR. a) Clonogenic survival of LL/2 cells treated with dose range of MMAE overnight and then re-plated in drug free media for 7-10 days to allow for visualized colonies to form. Colony number normalized to control untreated cells and plotted as mean fractional survival ± SEM, n=3. Statistical significances calculated using one-way ANOVA with Tukey's multiple comparisons test. **b)** Cell cycle analysis of MMAE treated LL/2 cells, mean ± SD, n=2. Results representative of 2 independent experiments. **c)** Clonogenic survival of LL/2 cells treated with MMAE and 2 Gy (SF2). Cell viability normalized to non-irradiated cells for each drug condition and plotted as mean fractional survival ± SEM, n=6. Statistical significances calculated using one-way ANOVA with Sidak's multiple comparisons test. **d)** Tumor volumes (n=10) and mouse body weights (n=5) of mice from experiments in **Fig. 3e.** Tumor volumes plotted as mean volume of surviving mice ± SEM. Mouse body weight were normalized to each mouse's weight on day 0, i.e. tumor implantation. Data plotted as mean fractional body weight ± SEM. **e)** Mouse body weights of mice (n=5) from experiments in **Fig. 4h** were normalized to each mouse's weight on day of tumor implantation. Data is plotted as individual normalized body weights. **f)** Mouse body weights of mice from experiments in **Fig. 5e, f** were normalized to each mouse's weight on day of tumor implantation. Data is plotted as mean fractional body weight ± SEM, anti-PD-1 alone, ACPP-MMAE ± IR (n=5), control IgG (n=6), ADC ± IR (n=7). Source data are provided in Source Data file.

Supplementary Figure 10

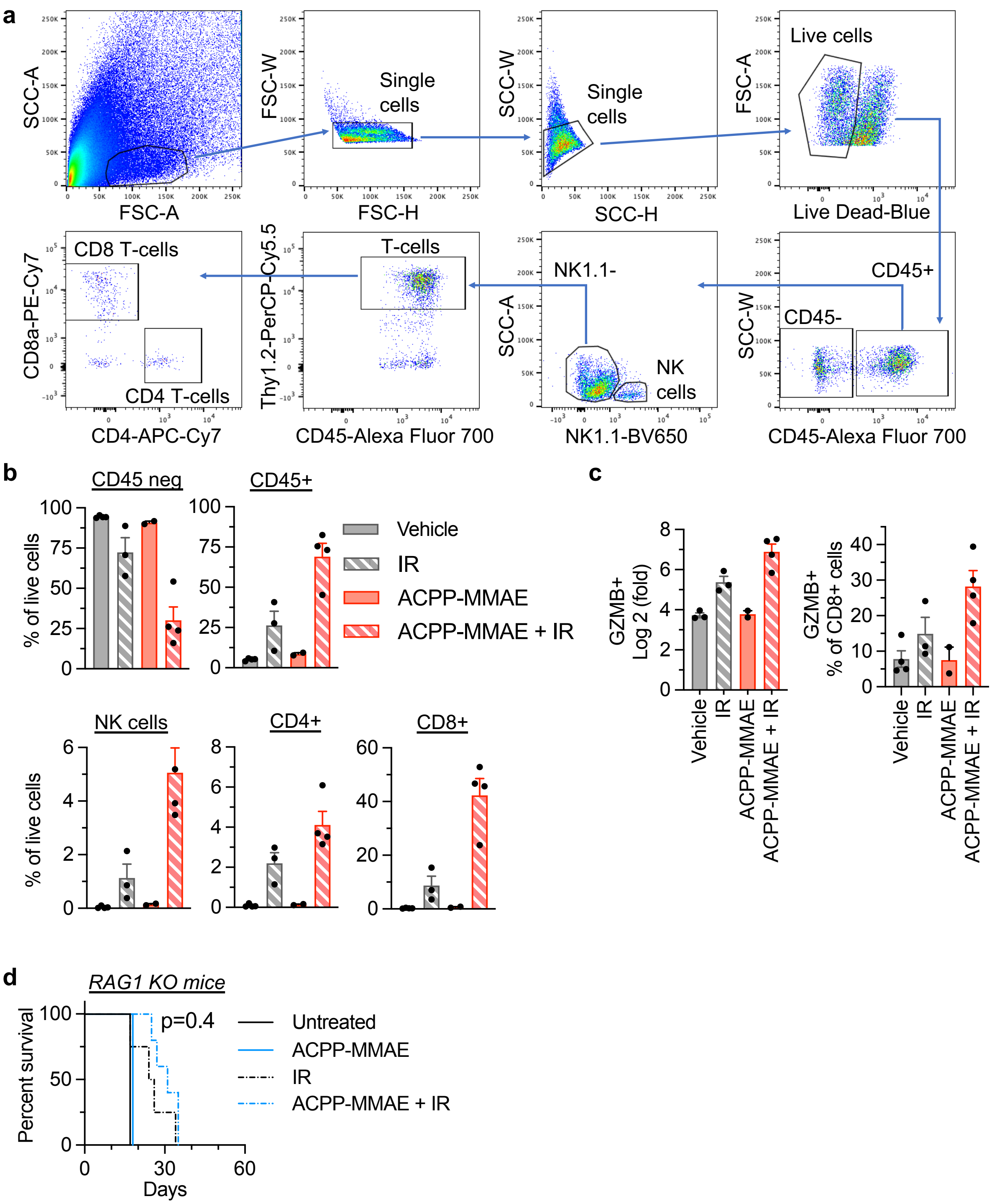

Supplementary Figure 10: Tumor immune infiltrate characterization and necessity. a) Representative flow cytometry plots to gate immune cells quantitated in **Fig. 4a, b, 4d** & **Supplementary Fig. 10b, c**. **b)** Quantification of tumor immune cell identified by flow cytometry on **Fig. 4a**. B16 tumors implanted on day 0 were treated with ACPP-MMAE and IR starting on day 9. Tumors harvested on day 19 and immune cells characterized by flow cytometry. **c)** Tumor granzyme B expression. Characterized CD8+ T-cells were further gated for granzyme-B staining (Clone:GB11). In addition, RNA levels of tumor granzyme-B was analyzed by qPCR. For **Supplementary Fig. 10b & c**, vehicle, ACPP-MMAE + IR, n=4; IR n=3 tumors; ACPP-MMAE, n=2. **d)** Efficacy of MMAE and IR in B16 tumors in RAG1 KO mice. Mice implanted with tumor cells on day 0 were injected with 10 nmoles ACPP-MMAE and 5 Gy as in **Fig. 3e** starting on day 3. Mouse survival plotted and statistical significance (IR vs ACPP-MMAE + IR) calculated using Log-rank (Mantel-Cox) test. For untreated and ACPP-MMAE alone, n=2 mice; IR, n=4 mice; ACPP-MMAE + IR, n=5 mice. Source data are provided in Source Data file.
