# Supplementary material for: Monomethyl auristatin antibody and peptide drug conjugates for trimodal cancer chemo-radio-immunotherapy
Source: Nat Commun. 2022 Jul 5;13:3869. doi: 10.1038/s41467-022-31601-z (PMC9256669; doi:10.1038/s41467-022-31601-z)
Supplement: Supplementary file 3 — Description of Additional Supplementary Files [file 41467_2022_31601_MOESM3_ESM.pdf]

Supplementary Data 1: Complete NanoString expression data of Figure 1b. Untreated control (n=2) and 5 nM MMAE treated (n=3) B16 cells were harvested at 72 hrs. RNA was collected and analyzed using NanoString PanCancer IO 360 Panel. Individual normalized RNA expression values shown for each gene.

Supplementary Data 2: Complete NanoString expression data of Figure 4c. B16 tumors from control (untreated), IR, ACPP-MMAE or ACPP-MMAE + IR treated mice (n=3 tumors/group) were harvested. RNA was collected and analyzed using NanoString nCounter PanCancer Mouse Immune Profiling panel. Individual normalized RNA expression values shown for each gene.
