# Supplementary material for: Monomethyl auristatin antibody and peptide drug conjugates for trimodal cancer chemo-radio-immunotherapy
Source: Nat Commun. 2022 Jul 5;13:3869. doi: 10.1038/s41467-022-31601-z (PMC9256669; doi:10.1038/s41467-022-31601-z)
Supplement: Supplementary file 6 — Reporting Summary [file 41467_2022_31601_MOESM6_ESM.pdf]

nature portfolio

Corresponding author(s): Sunil J Advani

Last updated by author(s): Jun 8, 2022

# Reporting Summary

Nature Portfolio wishes to improve the reproducibility of the work that we publish. This form provides structure for consistency and transparency in reporting. For further information on Nature Portfolio policies, see our Editorial Policies and the Editorial Policy Checklist.

## Statistics

For all statistical analyses, confirm that the following items are present in the figure legend, table legend, main text, or Methods section.

| n/a | Confirmed | |
|---|---|---|
| ☐ | ☒ | The exact sample size (*n*) for each experimental group/condition, given as a discrete number and unit of measurement |
| ☐ | ☒ | A statement on whether measurements were taken from distinct samples or whether the same sample was measured repeatedly |
| ☐ | ☒ | The statistical test(s) used AND whether they are one- or two-sided<br>*Only common tests should be described solely by name; describe more complex techniques in the Methods section.* |
| ☒ | ☐ | A description of all covariates tested |
| ☐ | ☒ | A description of any assumptions or corrections, such as tests of normality and adjustment for multiple comparisons |
| ☐ | ☒ | A full description of the statistical parameters including central tendency (e.g. means) or other basic estimates (e.g. regression coefficient) AND variation (e.g. standard deviation) or associated estimates of uncertainty (e.g. confidence intervals) |
| ☐ | ☒ | For null hypothesis testing, the test statistic (e.g. *F*, *t*, *r*) with confidence intervals, effect sizes, degrees of freedom and *P* value noted<br>*Give P values as exact values whenever suitable.* |
| ☒ | ☐ | For Bayesian analysis, information on the choice of priors and Markov chain Monte Carlo settings |
| ☒ | ☐ | For hierarchical and complex designs, identification of the appropriate level for tests and full reporting of outcomes |
| ☒ | ☐ | Estimates of effect sizes (e.g. Cohen's *d*, Pearson's *r*), indicating how they were calculated |

*Our web collection on statistics for biologists contains articles on many of the points above.*

## Software and code

Policy information about availability of computer code

| | |
|---|---|
| Data collection | CyTOF data collection used CyTOF software, version 7.0.8493 and MATLAB, version 9.5 |
| Data analysis | Data and statistical analyses were performed using GraphPad Prism software, version 9.1.2.<br>Cell Cycle and Flow cytometry data was analyzed using FlowJo software, version 10.6.2.<br>NanoString data were analyzed by The Advanced Analysis module of the nSolver software, version 4.0.<br>qPCR data were analyzed using the Bio-Rad CFX Maestro software, version 1.1.<br>Fluorescent image intensity was quantitated using Image J software, version 1.53i. |

For manuscripts utilizing custom algorithms or software that are central to the research but not yet described in published literature, software must be made available to editors and reviewers. We strongly encourage code deposition in a community repository (e.g. GitHub). See the Nature Portfolio guidelines for submitting code & software for further information.

## Data

Policy information about availability of data

All manuscripts must include a data availability statement. This statement should provide the following information, where applicable:

- Accession codes, unique identifiers, or web links for publicly available datasets
- A description of any restrictions on data availability
- For clinical datasets or third party data, please ensure that the statement adheres to our policy

All data reported in this work are available within the Article, Supplementary Information, Supplementary Data, or Source Data file.

## Human research participants

Policy information about studies involving human research participants and Sex and Gender in Research.

| | |
|---|---|
| Reporting on sex and gender | *Use the terms sex (biological attribute) and gender (shaped by social and cultural circumstances) carefully in order to avoid confusing both terms. Indicate if findings apply to only one sex or gender; describe whether sex and gender were considered in study design whether sex and/or gender was determined based on self-reporting or assigned and methods used. Provide in the source data disaggregated sex and gender data where this information has been collected, and consent has been obtained for sharing of individual-level data; provide overall numbers in this Reporting Summary. Please state if this information has not been collected. Report sex- and gender-based analyses where performed, justify reasons for lack of sex- and gender-based analysis.* |
| Population characteristics | *Describe the covariate-relevant population characteristics of the human research participants (e.g. age, genotypic information, past and current diagnosis and treatment categories). If you filled out the behavioural & social sciences study design questions and have nothing to add here, write "See above."* |
| Recruitment | *Describe how participants were recruited. Outline any potential self-selection bias or other biases that may be present and how these are likely to impact results.* |
| Ethics oversight | *Identify the organization(s) that approved the study protocol.* |

Note that full information on the approval of the study protocol must also be provided in the manuscript.

# Field-specific reporting

Please select the one below that is the best fit for your research. If you are not sure, read the appropriate sections before making your selection.

☒ Life sciences ☐ Behavioural & social sciences ☐ Ecological, evolutionary & environmental sciences

For a reference copy of the document with all sections, see nature.com/documents/nr-reporting-summary-flat.pdf

# Life sciences study design

All studies must disclose on these points even when the disclosure is negative.

| | |
|---|---|
| Sample size | No specific sample size calculation was performed. Mouse numbers used were empirically derived from the literature published in murine cancer models including our own prior work with radiosensitizing drugs tested in cell culture and murine models:<br>Buckel, et al, Cancer Research 2015, PMID: 25681274<br>Adams et al, Nature Communications 2016, PMID: 27698471<br>Hingorani et al Molecular Cancer Therapeutics 2020, PMID: 31597712<br>Hingorani et al Biomaterial 2020, PMID: 32304937<br>Based on preliminary studies with MMAE and radiotherapy in syngeneic mouse cancer models, the effect size was defined and we used biologically independent triplicates for non-therapy studies, 5 mice per group were reproducible in follow up repeat experiments and in across tumor models. |
| Data exclusions | No data were excluded from analyses |
| Replication | Reproducibility was rigorously addressed by complementary approaches.<br>1) For human xenograft studies, an isogeneic therapy resistant tumor line was tested and validated our hypothesis (CAL27 WT, CAL27 PIK3CA H1047R). In total, the human xenograft experiments were performed 3 independent times.<br>2) For syngeneic tumor models, 6 different tumor models (LL/2, B16, MC38, MOC2, MOC1, 4MOSC1) were tested and multiple models independently showed similar results across assays.<br>3) For drug targeting, 2 different classes of drug delivery platforms were used (antibody and cell penetrating peptides) and independently showed similar results across assays (tumor regression, tumor immune cell infiltration, necessity of CD8 T cells for durable tumor control and potentiation with immune checkpoint in. |

| | |
|---|---|
| | To ensure reproducibility, experiments were iteratively repeated in tumor models and confirmed the effectiveness of MMAE radiosensitization compared to monotherapies or untreated control tumors. Reproducibility was addressed multiple ways including 1) testing in different syngeneic tumor models LL/2, B16, MC38, 4MOSC1), 2) testing different MMAE delivery platforms (antibody or cell penetrating peptide), 3) testing adaptive immunity necessity genetically (RAG1 KO) and pharmacologically (anti-CD8), and activation of anti-tumor immune responses (NanoString and flow cytometry). These attempts at replication were successful. |
| Randomization | For animal studies, mice were randomly assigned to treatment groups on day 1 of treatment.<br>For cell culture studies involving individual plates, plates were randomly placed in treatment groups.<br>For cell culture studies involving 96 well plates, drug dosing proceeded in logical fashion from left to right to minimize drug dosing errors. |
| Blinding | For cell culture and mice studies, samples were coded by number to minimize bias.<br>For pathologic examination, pathologist was blinded to treatments of tissues to minimize bias. |

# Reporting for specific materials, systems and methods

We require information from authors about some types of materials, experimental systems and methods used in many studies. Here, indicate whether each material, system or method listed is relevant to your study. If you are not sure if a list item applies to your research, read the appropriate section before selecting a response.

## Materials & experimental systems

| n/a | Involved in the study |
|---|---|
| ☐ | ☒ Antibodies |
| ☐ | ☒ Eukaryotic cell lines |
| ☒ | ☐ Palaeontology and archaeology |
| ☐ | ☒ Animals and other organisms |
| ☒ | ☐ Clinical data |
| ☒ | ☐ Dual use research of concern |

## Methods

| n/a | Involved in the study |
|---|---|
| ☒ | ☐ ChIP-seq |
| ☐ | ☒ Flow cytometry |
| ☒ | ☐ MRI-based neuroimaging |

## Antibodies

| | |
|---|---|
| Antibodies used | For immunoblotting antibodies were obtained from Cell Signaling Technology:<br>HER3/ErbB3, Cell Signaling Technology, catalog number 12708, clone D22C5, lot number 4.<br>GAPDH, Cell Signaling Technology, catalog number 2118, clone 14C10, lot number 10.<br><br>All flow cytometry antibodies from BioLegend, San Diego, CA:<br>For mouse experiments, we used CD45 (30-F11) (1:100), CD90.2 (30-H12) (1:200) , CD8a (53-6.7) (1:100), CD4 (RM4-4) (1:400), NK1.1 (PK136) (1:100); PD-1 (29F.1A12) (1:100)<br><br>The antibodies for CyTOF are the following:<br>B220 (RA3-6B2), CD117(2B8), CD11c (N418), CD25 (3C7), CD4 (RM4-5), CD45 (30-F11), CD8a (53-6.7), MHC-II (M5/114.15.2), NKP46 (29A1.4), TCRb (H57-597) are from Fluidigm.<br>CD103 (2E7), CD115 (AFS98), CD11b (M1/70), CD19 (6D5), CD23 (B3B4), CD3 (145-2C11), CD64 (X54-5/7.1), F4/80 (BM8), FCeRI (Mar-1), FR4 (TH6), Ly6C (HK1.4), Ly6G (1A8), NK1.1 (PK136) are from Biolegend.<br>Siglec-F (1RNM44N) is from ebioscience;<br>All antibodies were used at a 1:100 dilution.<br><br>Therapeutic antibodies from Bio X Cell (West Lebanon, NH, USA): PD-1 antibody (clone J43, catalog #BE0033-2), (isotype antibody (catalog # BE0091) and CD8 depletion antibody (Clone YTS 169.4, catalog #BE0117). CDX3379 is provided by Celldex Therapeutics for both therapeutic purpose and flow cytometry. This antibody is designed to block the activity of ErbB3 (HER3) in both human and mouse. Cetuximab was clinical grade Erbitux from Lilly Medical and obtained from UCSD Moores Pharmacy. This antibody is designed to block the activity of ErbB1 (EGFR) in human cells. |
| Validation | All commercial antibodies used were validated by the supplier and were checked in the lab by comparing to the manufacturer's or in-house results.<br><br>1) For Cell Signaling Technology antibodies to HER3/ErbB3 and GAPDH: The CST Antibody Performance Guarantee; CST validates all antibodies, in-house, in multiple research applications, https://www.cellsignal.com/about-us/cst-antibody-performance-guarantee.<br><br>2) For BioLegend antibodies to CD45 (30-F11), CD90.2 (30-H12), CD8a (53-6.7), CD4 (RM4-4), NK1.1 (PK136), PD-1 (29F.1A12), CD103 (2E7), CD115 (AFS98), CD11b (M1/70), CD19 (6D5), CD23 (B3B4), CD3 (145-2C11), CD64 (X54-5/7.1), F4/80 (BM8), FCeRI (Mar-1), FR4 (TH6), Ly6C (HK1.4), Ly6G (1A8), NK1.1 (PK136): Statement from BioLegend: BioLegend antibodies undergo an extensive series of testing to ensure quality at every step in the manufacturing process, as well as maintaining quality after the sale.<br><br>3) For Bio X Cell antibodies PD-1 antibody (clone J43), CD8 depletion antibody (Clone YTS 169.4): Statement from Bio X Cell: Our InVivoPlus™ antibodies feature all the great qualities of our InVivoMab™ antibodies. The InVivoPlus™ versions of our products are structurally and functionally identical to the InVivoMab™ versions with the added benefit of additional QC measures. InVivoPlus™ |

antibodies are screened for murine pathogens using ultrasensitive qPCR, screened for protein aggregation via dynamic light scattering, feature advanced binding validation via flow cytometry, ELISA, and/or Western blot, and are guaranteed to contain less than 1 endotoxin unit per milligram. Our InVivoPlus™ line of antibodies are designed to exceed the strict demands and rigorous standards required for in vivo work at any research organization.

4) For Fluidigm antibodies to B220 (RA3-6B2), CD117(2B8), CD11c (N418), CD25 (3C7), CD4 (RM4-5), CD45 (30-F11), CD8a (53-6.7), MHC-II (M5/114.15.2), NKP46 (29A1.4), TCRb (H57-597): please see antibody information from this link: https://www.fluidigm.com/citations/antibody-validation, and antibody titration information from this link: https://www.fluidigm.com/citations/titration-of-mass-cytometry-reagents.

## Eukaryotic cell lines

Policy information about cell lines and Sex and Gender in Research

| | |
|---|---|
| Cell line source(s) | Human head and neck cancer cell lines (CAL27, Detroit 562), murine lung (LL/2) and murine melanoma (B16) were obtained from American Type Culture Collection. Murine colorectal (MC38) cancer cell lines were obtained from Kerafast. Murine head and neck MOC1 and MOC2 were generously provided by Dr. R. Uppaluri. CAL27 PIK3CA H1047R expressing cells and 4MOSC1 cells were previously characterized: DOI: 10.1038/s41467-019-13471-0, DOI:10.1038/s41467-021-22619-w. |
| Authentication | Cells were used at limited passage and were not authenticated. |
| Mycoplasma contamination | All cell lines used tested negative for Mycoplasma contamination. |
| Commonly misidentified lines (See ICLAC register) | None used. |

## Animals and other research organisms

Policy information about studies involving animals; ARRIVE guidelines recommended for reporting animal research, and Sex and Gender in Research

| | |
|---|---|
| Laboratory animals | 1) Athymic nu/nu mice, female, 4-6 week old<br>2) C57BL/6 albino mice, female, 6 week old<br>3) NOD/SCID, female, 6 week old<br>4) RAG1 KO mice, Rag1tm1Mom , female, 6 week old<br><br>Animal vivarium conditions - light/dark cycle: 12 hours; temp: 18-23 degrees C, humidity: 40-60% |
| Wild animals | Study did not involve wild animals. |
| Reporting on sex | All reported studies were performed in female mice that facilitated animal handling, randomization and cage re-assortment. |
| Field-collected samples | Study did not involve field-collected samples. |
| Ethics oversight | All animal work was done in compliance with the University of California San Diego Institutional Animal Use and Care Committee. |

Note that full information on the approval of the study protocol must also be provided in the manuscript.

## Flow Cytometry

### Plots

Confirm that:

- [x] The axis labels state the marker and fluorochrome used (e.g. CD4-FITC).
- [ ] The axis scales are clearly visible. Include numbers along axes only for bottom left plot of group (a 'group' is an analysis of identical markers).
- [x] All plots are contour plots with outliers or pseudocolor plots.
- [x] A numerical value for number of cells or percentage (with statistics) is provided.

### Methodology

| | |
|---|---|
| Sample preparation | At harvest, tumors were isolated, minced, and re-suspended in FBS-free DMEM media supplemented with components of MACs tumor dissociation kit. Tissues were incubated for 15 minutes at 37°C and mechanically digested using the gentle MACs Octo Dissociator. Tissue suspensions were washed with fresh media and passed through a 100-µm strainer. Samples were split in half for immediate staining or stimulated with the Biolegend Cell Activation Cocktail (with Brefeldin A) for 3 hours. Samples were washed with PBS and immediately processed for live/dead cell discrimination using BD LIVE/DEADTM Fixable Blue Dead Cell Stain Kit. Cells were washed and stained for surface markers for 30 minutes at 4° C. |
| Instrument | BD LSRFortessa |

| | |
|---|---|
| Software | FlowJo |
| Cell population abundance | Cells were not sorted in this study. |
| Gating strategy | Single cells were gated from FSC/SSC (height/width) and live/dead cells were discriminated using BD LIVE/DEADTM Fixable Blue Dead Cell Stain Kit. All flow cytometry data acquisition was done using BD LSRFortessa and analyzed using FlowJo software. Immune cells were identified by the following characteristics: cytotoxic T cells (CD45+Thy1.2+CD8+), helper T cells (CD45+Thy1.2+CD4+), NK cells (CD45+Thy1.2-NK1.1+). |

☒ Tick this box to confirm that a figure exemplifying the gating strategy is provided in the Supplementary Information.
